# Kinase Inhibitor VvBKI1 Interacts with Ascorbate Peroxidase VvAPX1 Promoting Plant Resistance to Oomycetes

**DOI:** 10.3390/ijms24065106

**Published:** 2023-03-07

**Authors:** Junli Lv, Wei Wu, Tao Ma, Bohan Yang, Asaf Khan, Peining Fu, Jiang Lu

**Affiliations:** Center for Viticulture and Enology, School of Agriculture and Biology, Shanghai Jiao Tong University, Shanghai 200240, China

**Keywords:** VvBKI1, VvAPX1, oomycete, downy mildew, resistance

## Abstract

Downy mildew caused by oomycete pathogen *Plasmopara viticola* is a devastating disease of grapevine. *P. viticola* secretes an array of RXLR effectors to enhance virulence. One of these effectors, PvRXLR131, has been reported to interact with grape (*Vitis vinifera*) BRI1 kinase inhibitor (VvBKI1). BKI1 is conserved in *Nicotiana benthamiana* and *Arabidopsis thaliana*. However, the role of VvBKI1 in plant immunity is unknown. Here, we found transient expression of *VvBKI1* in grapevine and *N. benthamiana* increased its resistance to *P. viticola* and *Phytophthora capsici*, respectively. Furthermore, ectopic expression of VvBKI1 in Arabidopsis can increase its resistance to downy mildew caused by *Hyaloperonospora arabidopsidis*. Further experiments revealed that VvBKI1 interacts with a cytoplasmic ascorbate peroxidase, VvAPX1, an ROS-scavenging protein. Transient expression of *VvAPX1* in grape and *N. benthamiana* promoted its resistance against *P. viticola*, and *P. capsici*. Moreover, *VvAPX1* transgenic Arabidopsis is more resistant to *H. arabidopsidis*. Furthermore, both VvBKI1 and VvAPX1 transgenic Arabidopsis showed an elevated ascorbate peroxidase activity and enhanced disease resistance. In summary, our findings suggest a positive correlation between APX activity and resistance to oomycetes and that this regulatory network is conserved in *V. vinifera*, *N. benthamiana*, and *A. thaliana*.

## 1. Introduction

Plants are constantly challenged by different pathogens. For this reason, plants have evolved a sophisticated immune system to ward off pathogens and to ensure their survival. To restrict pathogens, plants recognize conserved pathogen-associated molecular patterns (PAMPs) via pattern recognition receptors (PRRs) by activating PAMP-triggered immunity (PTI). To evade recognition from the plant PRRs, pathogens have evolved effectors, which are delivered into the host to suppress PTI. Plants in turn have evolved resistance proteins to recognize effector proteins either directly or indirectly [1].

Grape (*Vitis vinifera* L.) is an economically important fruit crop cultivated worldwide. However, its yield and quality are largely affected by a number of diseases, such as downy mildew (DM) caused by *Plasmopara viticola*. *P. viticola* usually infects the green tissues of grapevines including leaves, young fruits, young shoots, and tendrils [2]. Similar to other pathogens, *P. viticola* also has evolved a repertoire of effector proteins known as PvRXLRs to promote plant colonization and evade PTI. However, some of them can be recognized by the intracellular resistance proteins that result in effector-triggered immunity (ETI) [1]. During this host–pathogen interaction, reactive oxygen species (ROS) is produced in both PTI and ETI [1,3].

Our previous study shows that *P. viticola* effector PvRxLR131 targets VvBKI1(BRI1 kinase inhibitor) to suppress plant immunity. When BKI1 was silenced in *N. benthamiana*, PvRxLR131 could not suppress plant resistance to *P. capsici* [4]. BKI1 belongs to the Membrane-Associated Kinase Regulator (MAKR) family, which is characterized by intrinsically disordered. BKI1 and other members of this family evolved from a common ancestor [5,6]. BKI1 has been reported as a negative regulator of brassinosteroid (BR) signaling pathway in Arabidopsis. Application of BR results in dissociation of AtBKI1 from the plasma membrane into the cytosol, thereby relieving the inhibition of AtBKI1 on transphosphorylation between AtBRI1 and its coreceptor AtBAK1 [7]. It is reported that [KR][KR] repeat (arginine (K); lysine (R)) in the middle of AtBKI1 is key for its localization [8]. BKI1 contains a BRI1-interacting motif (BIM) in C-terminal which interacts with BRI1 kinase domain [9]. The phosphorylated AtBKI1 competes with 14-3-3κ in BRI1 EMS SUPPRESSOR1 (AtBES1) and helps to accumulate AtBES1 in the nucleus to enhance BR signaling [10]. AtBKI1 can also regulate pedicel orientation by interacting with another important LRR-RLK, ERECTA (ER) [11]. Furthermore, PUB30, a U-box E3 ubiquitin ligase, was found to interact with BKI1, which resulted in its degradation. Arabidopsis plants overexpressing AtBKI1 were found more salt-tolerant compared with the wild-type [12]. The C4 protein of tomato leaf curl Yunnan virus (TLCYnV) was found to interact with BKI1 in tobacco which inhibits the dissociation of ER and BKI1 complex thereby disrupting the activation of mitogen-activated protein kinase (MAPK) cascades subsequently resulting in an improved infection [13].

ROS, an oxygen containing molecules with higher chemical reactivity [14], are widespread metabolites in all aerobic organisms. Elevated ROS is not only caused by biotic but also abiotic stresses [15]. As they can cause direct oxidative damage to DNA, lipids, and proteins, ROS were initially considered as bad substances [16]. Research findings in the last two decades have demonstrated ROS as an important regulatory molecule in pollen tube elongation, root hair growth, cell polarity, and Casparian strip formation [17,18,19]. The maintenance of redox homeostasis plays key roles. In plants, the main forms of reactive oxygen species are singlet oxygen (^1^O_2_), superoxide anion (O_2_·−), hydrogen peroxide (H_2_O_2_), and hydroxyl radicals (HO·) [14]. To avoid toxicity, ROS concentrations must be maintained at appropriate levels by ROS-producing and scavenging enzymes. Major enzymatic ROS-scavenging mechanisms in plants include superoxide dismutase (SOD), ascorbate peroxidase (APX), glutathione peroxidase (GPX), and catalase (CAT). SODs provide primary defense against ROS and dismutate superoxide to H_2_O_2_, while APX, GPX, and CAT subsequently detoxify H_2_O_2_. Among these enzymes, APX requires ascorbate as a substrate [20], and has been widely studied in Arabidopsis [21].

Ascorbate peroxidases (APXs) are heme peroxidases that are involved in the removal of hydrogen peroxide in different subcellular compartments with concomitant ascorbate cycling. Ascorbate peroxidases are members of peroxidase superfamily I and are abundantly present in plants, fungi, and bacteria [22,23]. In Arabidopsis, APXs are classified as cytosolic (APX1, APX2, and APX6), peroxisomal (APX3, APX4, and APX5), and chloroplastic (stromal APX and thylakoid-bound APX), based on their subcellular localization. AtAPX1 has been reported to play a dual role, i.e., the dimeric form of AtAPX1 acts as peroxidases, while the high-molecular weight (HMW) form functions as a chaperone molecule. This dual nature of AtAPX1 was strongly dependent on its structural conformation and in vivo experiments suggested that the conformational changes in AtAPX1 are regulated by abiotic stress [24]. Similarly, AtAPX1 has been reported to play an important role in response to plant abiotic stresses. For example, APX1 deficient Arabidopsis plants were found more susceptible to light stress or a combination of drought and heat stress and more H_2_O_2_ accumulation was recorded under an abiotic environment in Arabidopsis [25,26]. However, *apx1* mutant Arabidopsis plants were more resistant to selenium (Se) and lead (Pb) [27,28]. APX1 has also been found to play an important role in the growth and development of plants. The number of lateral roots were strongly affected in *apx1* Arabidopsis mutant plants when they were treated with auxin [29]. Increased expression of GhAPX1 improved fiber cell elongation in cotton [30]. Functions of the APX genes in grape remain to be determined.

The aim of the present study was to investigate the role of VvBKI1 in resistance against grapevine downy mildew and its correlation to VvAPX1 against *P. viticola* and other oomycete pathogens in plant immunity.

## 2. Results

### 2.1. VvBKI1 Promotes Plant Resistance to Oomycetes

**Figure 1:**
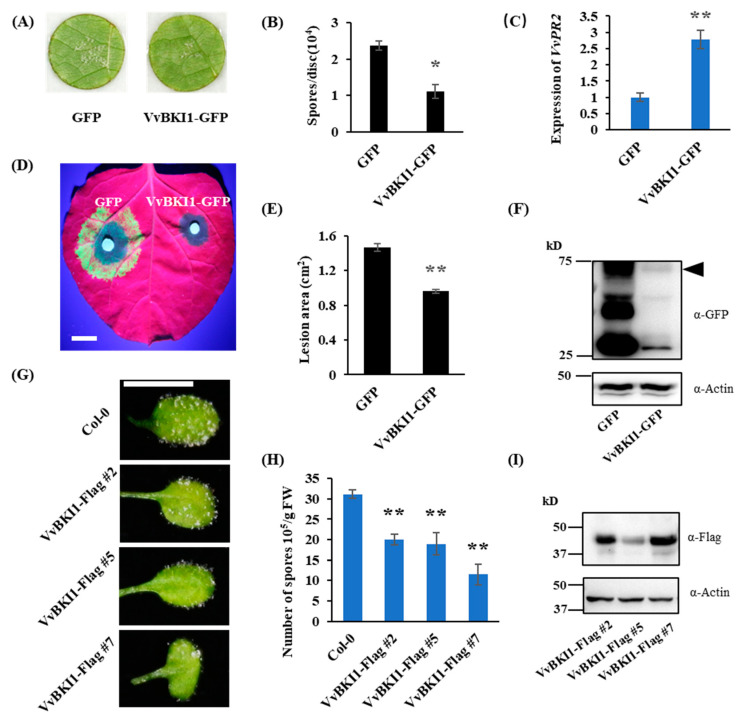
VvBKI1 positively regulate plant immunity. (**A**,**B**) *Plasmopara viticola* was inoculated at 4 days after agroinfiltration. Count of spores and photo were taken at 7 days after inoculation. (**C**) Expression level of pathogen-related gene *PR2* at 3 dpi. (**D**) *Phytophthora capsici* was inoculated onto *N. benthamiana* leaves at 24 h after agroinfiltration. Photo was taken at 60 hpi under UV. Scale bar = 1 cm. (**E**) Lesion areas of *P. capsici* after transient expression of VvBKI1. Data are showed as the mean ± SD (*n =* 11). (**F**) Western blot of VvBKI1 in *N. benthamiana* with anti-GFP antibody, at 2 days after agroinfiltration. Triangle represents VvBKI1-GFP. (**G**) Phenotype of two-week-old VvBKI1 transgenic Arabidopsis inoculated with *Hyaloperonospora arabidopsidis*. Photo was taken at 4 dpi. Scale bar = 0.5 cm. (**H**) Two-week-old VvBKI1 transgenic Arabidopsis were inoculated with *H. arabidopsidis*. Spores were counted at 4 dpi. Data are means ± SD from 3 biological replicates. (**I**) Western blot of VvBKI1 transgenic Arabidopsis with anti-Flag antibody. * represents significant difference (*p* < 0.05, Student’s *t* test). ** represents extremely significant difference (*p* < 0.01, Student’s *t* test).

To understand the role of *VvBKI1* in plant immunity, VvBKI1-GFP and GFP were transiently expressed in grape leaves. The corresponding leaf discs were inoculated with *Plasmoapora viticola* at 4 days after agroinfiltration and spores were counted after 7 dpi (days post-inoculation). The results indicated that spore formation was significantly reduced on leaves expressing VvBKI1-GFP compared with leaves expressing GFP. The result suggests that VvBKI1 can increase resistance of grapevine to *P. viticola* (Figure 1A,B). To check the expression level of *VvBKI1,* total RNA was extracted from the agroinfiltrated leaves and qRT-PCR was carried out with *VvBKI1* specific primers. The results indicated that the transcript levels of *VvBKI1* were increased by 29-fold compared with control (Supplementary Figure S1A). We also analyzed the expression level of a pathogen-related gene *VvPR2*, which was indeed significantly increased by VvBKI1, after *P. viticola* inoculation (Figure 1C).

To know if VvBKI1 can increase resistance to other oomycetes pathogens, we transiently expressed VvBKI1 in leaves of *Nicotiana benthamiana* and the corresponding leaves were inoculated with *Phytophthora capsici* 24 h post-agroinfiltration. The results indicated that leaves expressing VvBKI1 showed significantly smaller lesions compared with the control (Figure 1D,E) suggesting that VvBKI1 can positively regulate *N. benthamiana* resistance to the oomycete pathogen *P. capsici*. Protein expression of VvBKI1 in the infiltrated leaves was confirmed by Western blotting (Figure 1F). Similarly, we also tested the role of VvBKI1 response to *Hyaloperonospora arabidopsidis* Noco2 in Arabidopsis. Two-week-old VvBKI1 Arabidopsis transgenic lines were inoculated with *H. arabidopsidis* and spores were counted at 4 dpi. The result indicated that spore formation was significantly inhibited in VvBKI1 transgenic lines compared with wild-type (Figure 1F,G). VvBKI1 transgenic lines were confirmed by Western blot (Figure 1I). The above results suggest that VvBKI1 plays a positive role in resistance against oomycete pathogens.

### 2.2. VvAPX1 Can Interact with VvBKI1

During investigation of the mechanism how BKI1 promoting plant resistance to oomycete pathogen, an immunoprecipitation–mass spectrometry (IP-MS) assay was conducted using *N. benthamiana* to find the interacting protein of BKI1. One of the proteins identified through MS assay was NbAPX1 (Niben101Scf13258g00025.1). The peptide identified in IP-MS is listed in Supplementary Table S1.

**Figure 2:**
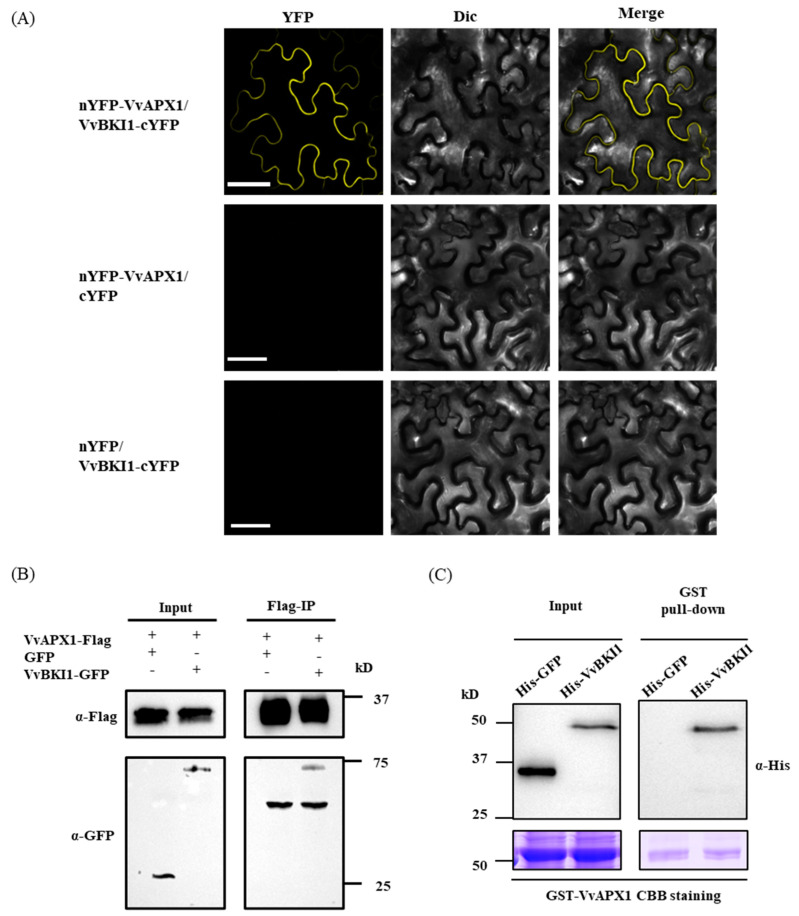
VvBKI1 interacts with VvAPX1 in vivo and in vitro. (**A**) BiFC assay indicates VvBKI1 interacting with VvAPX1 in vivo. nYFP-VvAPX1 and VvBKI1-cYFP was co-expressed for 48 h. Scale bar = 50 μm. (**B**) CoIP assay indicates that VvBKI1 interacting with VvAPX1 in vivo. (**C**) Pull-down assay indicates that VvBKI1 interacting with VvAPX1 in vitro. His-GFP was used as negative control. The Coomassie brilliant blue (CBB) staining shows equal loading.

Given that NbAPX1 is a potential interactor of NbBKI1, and that NbAPX1 is homologue of VvAPX1, it is possible that VvBKI1 might interact with VvAPX1. A subsequent BiFC experiment was therefore conducted. VvBKI1 was fused with C-terminal YFP (cYFP), while VvAPX1 fused with N-terminal YFP (nYFP). VvBKI1-cYFP and nYFP-VvAPX1 were co-expressed in *N. benthamiana*. YFP fluorescent signals were detected on the plasma membrane of *N. benthamiana* cells co-expressing VvBKI1-cYFP and nYFP-VvAPX1, indicating that VvBKI1 interacts with VvAPX1 in vivo (Figure 2A). The interaction was also confirmed by Co-immunoprecipitation (Co-IP) assay. VvAPX1-Flag was co-expressed with GFP or VvBKI1-GFP in *N. benthamiana*. Total protein was extracted 48 h post-agroinfiltration and incubated with anti-Flag magnetic beads. VvBKI1-GFP can be detected by Western blotting (Figure 2B). Furthermore, we performed GST pull-down assay with glutathione S-transferase (GST)-tagged VvAPX1 and His-tagged VvBKI1 synthesized from *Escherichia coli* (DE3). As shown in Figure 2C, His-VvBKI1 was pulled down by GST-VvAPX1, demonstrating that VvBKI1 interacts with VvAPX1 in vitro.

### 2.3. Characterization of VvAPX1

**Figure 3:**
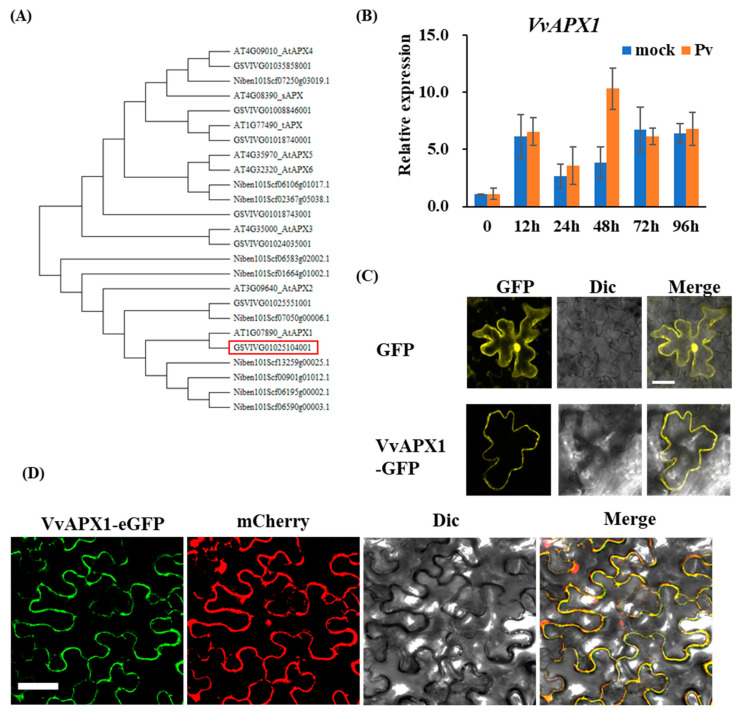
Phylogenetic, Transcript pattern, and subcellular analysis of VvAPX1. (**A**) Phylogenetic analysis of APX in *Vitis vinera*, *Nicotiana benthamiana*, and *Arabidopsis thaliana*. Red box represents VvAPX1. Phylogenetic tree was constructed using the neighbor-joining method with MEGA-X. (**B**) Transcript pattern of *VvAPX1* during *P. viticola* infection. The leaf discs of *Vitis vinifera* ‘Thompson Seedless’ were inoculated with *P. viticola*. Samples were harvested at 0, 12, 24, 48, 72, and 96 hpi for RNA extraction. (**C**) Subcellular localization of VvAPX1 in *V. vinera* ‘Thompson Seedless’. Scale bar = 10 μm. (**D**) Subcellular localization of VvAPX1 in *N. benthamiana.* Scale bar = 50 μm.

Phylogenetic analysis showed VvAPX1 and NbAPX1 are homologues of AtAPX1, which is a cytoplasmic ascorbate peroxidase (Figure 3A).

In this study, expression of *VvAPX1* in grapevine leaves was measured after *P. viticola* inoculation. Transcriptional expression of *VvAPX1* was enhanced by *P. viticola* infection after 12 hpi and peaked at 48 hpi, where the expression level was 3 times higher than it in uninoculated leaves (Figure 3B).

To further look into the subcellular localization of VvAPX1, we transiently expressed VvAPX1-GFP in leaves of grapes following agroinfiltration. GFP signals were mainly detected in the cytoplasm (Figure 3C) suggesting that VvAPX1 is localized to cytoplasm. Furthermore, when VvAPX1-GFP was co-expressed with mCherry, fluorescent signals of GFP and mCherry were merged in the cytoplasm (Figure 3D), suggesting that VvAPX1 is also a cytoplasmic ascorbate peroxidase.

### 2.4. VvAPX1 Positively Regulate Plant Resistance to Oomycetes

**Figure 4:**
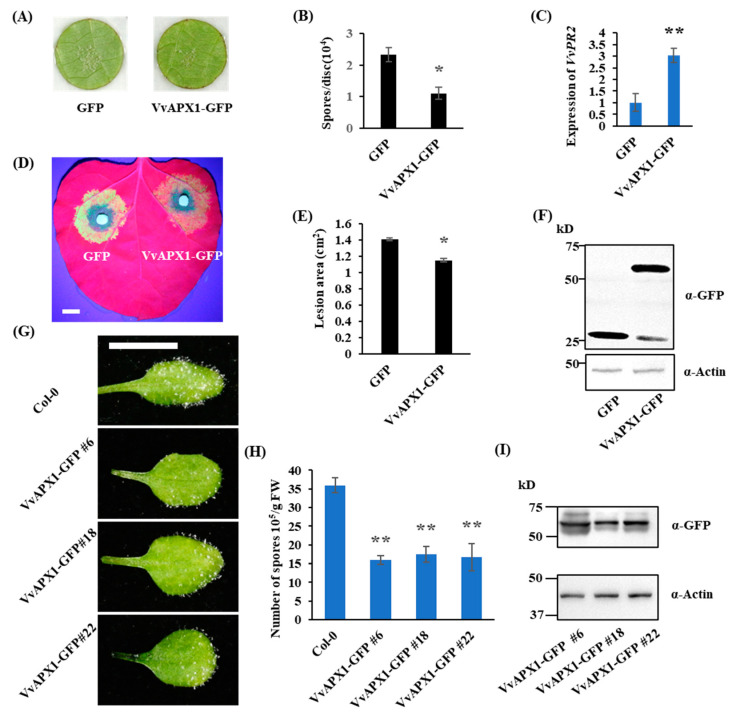
VvAPX1 positively regulate plant immunity. (**A**,**B**) *P. viticola* was inoculated at 4 days after agroinfiltration. Count of spores and photo were taken at 7 dpi. (**C**) Expression level of pathogen-related gene *PR2* was determined at 3 dpi. (**D**) *P. capsici* was inoculated onto *N. benthamiana* leaves at 24 h after agroinfiltration. Photo was taken at 60 hpi under UV. Scale bar = 1 cm. (**E**) Lesion area of each construct. Data are shown as the mean ± SD (*n =* 6). (**F**) Western blot of protein level of VvAPX1 in *N. benthamiana* with anti-GFP antibody, at 2 days after agroinfiltration. (**G**,**H**) Two-week-old Arabidopsis were inoculated with *H. arabidopsidis*. Photo was taken at 4 dpi. Scale bar = 0.5 cm. Spores were counted at 4 dpi. (**I**) Western blot of protein level of VvAPX1 in transgenic Arabidopsis with anti-GFP antibody. * represents significant difference (*p* < 0.05, Student’s *t* test). ** represents extremely significant difference (*p* < 0.01, Student’s *t* test).

After confirming the interaction between VvBKI1 and VvAPX1 we then asked whether VvAPX1 has some roles in plant immunity. *VvAPX1* was transiently overexpressed in grape leaves. A significant increase in the transcript levels of *VvAPX1* were observed 4 days after agroinfiltration (Supplementary Figure S1B). *P. viticola* was inoculated onto the leaf discs 4 days after agroinfiltration. Interestingly, overexpression of *VvAPX1* resulted in an increase of grape resistance to *P. viticola* (Figure 4A,B). We then looked for the expression level of a pathogen-related gene, *VvPR2*, which was also strongly induced upon overexpression of VvAPX1 (Figure 4C).

In a subsequent study, *VvAPX1* was transiently expressed in *N. benthamiana* leaves, followed by *P. capsici* inoculation 1 day after agroinfiltration. The results indicated that expression of VvAPX1 results in a significantly smaller lesion area compared with GFP, suggesting a positive role of VvAPX1 in resistance *N. benthamiana* against *P. capsici* (Figure 4D,E). Protein expression of VvAPX1 was confirmed by Western blotting with GFP antibody (Figure 4F).

Furthermore, we ectopically expressed VvAPX1 in Arabidopsis to evaluate its function in response to the oomycete pathogen *H. arabidopsidis*. Two-week-old Arabidopsis plants were inoculated with *H. arabidopsidis* and spores were counted at 4 dpi. Spore formation in transgenic plants were significantly reduced compared with wild-type (Figure 4G,H). These findings suggest that VvAPX1 can contribute to resistance of Arabidopsis to *H. arabidopsidis*. Protein expression of VvAPX1 in Arabidopsis transgenic plants was confirmed by Western blot with GFP antibody (Figure 4I).

All the above findings clearly demonstrate that VvAPX1 positively regulates plant resistance to oomycete pathogens.

### 2.5. Ectopical Expression of VvAPX1 and VvBKI1 Enhance Total APX Activity of Arabidopsis

**Figure 5:**
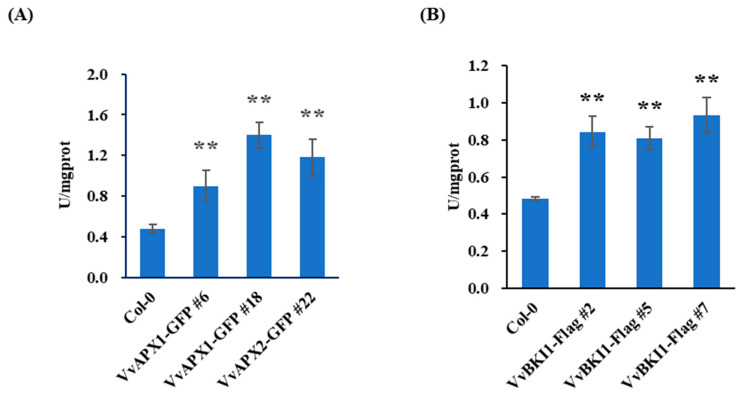
Total Ascorbate Peroxidase (APX) activity of VvAPX1 and VvBKI1 transgenic Arabidopsis. (**A**) Ectopic expression of VvAPX1 in Arabidopsis increases plant enzyme activity. (**B**) Ectopic expression of VvBKI1 in Arabidopsis increases plant enzyme activity. Total protein from rosette leaves of four-week-old Arabidopsis were used to check APX activity. Data are shown as means ± SD (*n =* 3). ** represents extremely significant difference (*p* < 0.01, Student’s *t* test).

Ascorbate peroxidase (APX) is an important reactive oxygen species (ROS)-scavenging enzyme. APX catalyzes the reaction of ascorbic acid (ASA) with hydrogen peroxide, transforming ascorbic acid to monodehydroascorbic acid (MDASA) to prevent oxidative damage. In this study we checked the APX activity of the *VvAPX1* in 4-week-old transgenic Arabidopsis seedlings. The active unit represents 1 mg of protein catalyzing 1 μmol ASA per minute in 1 mL reaction system. Total APX activity in the VvAPX1 transgenic Arabidopsis plants was increased to 2–3-fold compared with wild-type (Figure 5A), indicating that VvAPX1 can significantly increase the overall APX activity. We then asked whether VvBKI1 has an impact on the APX activity. For this purpose, VvBKI1 was ectopically expressed in Arabidopsis plants, and total APX activity was measured. The results indicated that APX activity increased to 2-fold in *VvBKI1* transgenic plants (Figure 5B).

### 2.6. Function of AtBKI1 and AtAPX1 in Resistance and APX Activity

**Figure 6:**
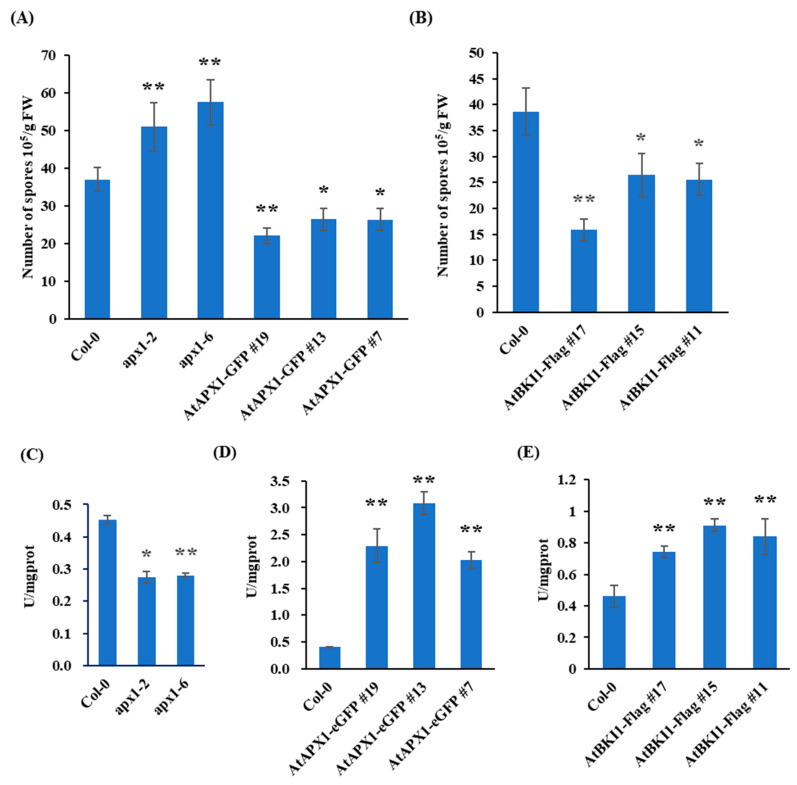
Function of AtBKI1 and AtAPX1 in Arabidopsis in resistance and APX activity. (**A**) Two-week-old AtBKI1 overexpression lines were inoculated with *H. arabidopsidis*. Spores were counted at 4 dpi. Data are means ± SD from 3 biological replicates. (**B**) Two-week-old AtAPX1 overexpression lines were inoculated with *H. arabidopsidis*. Spores were counted at 4 dpi. Data are means ± SD from 4 biological replicates. (**C**–**E**) Total APX activity of *apx1* mutants, Arabidopsis plants overexpressing AtAPX1 and AtBKI1. Data are shown as means ± SD (*n =* 3). * Represents significant difference (*p* < 0.05, Student’s *t* test). ** represents extremely significant difference (*p* < 0.01, Student’s *t* test).

To further understand their possible role in resistance of plants against pathogens we generated Arabidopsis plants overexpressing AtBKI1 and AtAPX1. ABKI1 and AtAPX1 protein levels in overexpressing Arabidopsis lines was confirmed through Western blot (Supplementary Figure S2A,B) while *apx1* mutant was identified by semi-RT PCR (Supplementary Figure S2C). We then inoculated two-week-old Arabidopsis plants with *H. arabidopsidis* and spores were counted at 4 dpi. The results indicated a significant reduction in spores on transgenic plants compared with wild-type. On the other hand, a significant increase in spores on the *apx1* mutant was observed. It is indicated that both AtAPX1 and AtBKI1 can enhance resistance of Arabidopsis to *H. arabidopsidis* (Figure 6A,B). We then detected their APX activity. The results indicated that the APX activity in *apx1* mutant plants was about 60% compared with wild-type (Figure 6C). This suggests AtAPX1 plays an important role in the APX activity of plants. As expected, overexpression of AtAPX1 and AtBKI1 also resulted in an increase in the APX activity (Figure 6D,E). These results indicate that APX activity may positively regulate resistance against oomycete pathogens.

## 3. Discussion

Diseases caused by oomycetes such as *Phytophthora infestans* [31], *Phytophthora cinnamomi* and *P. viticola* are responsible for huge economic loses annually [32,33]. Among them, downy mildew is devastative to grapes. The obligate biotrophic nature of *P. viticola* and the inefficiency of grapevine transformation limits our understanding of the underlying mechanism. In this study, we therefore took advantage of heterologous system, i.e., *H. arabidopsidis*–Arabidopsis and *P. capsici*–*N. benthamiana* interaction, to understand the role of VvBKI1 and VvAPX1 in plant—oomycete pathogen interactions.

To look for possible homologue of APX in grapes, we used blast tool of Phytozome (https://phytozome-next.jgi.doe.gov/, accessed on 7 November 2021) using APX1 (Niben101Scf13258g00025.1) of *N. benthamiana* as query. Two proteins, GSVIVT01025104001 and GSVIVT01025551001 classified as L-ascorbate peroxidase 2 were identified as possible APX1 homologues in grape. Our phylogenetic analysis indicated that GSVIVT01025104001 is closely related to AtAPX1, we therefore designated GSVIVT01025104001 as VvAPX1 and GSVIVT01025551001 as VvAPX2. Since VvAPX2 was not induced during *P. viticola* infection (Supplementary Figure S3). We therefore focused on VvAPX1 in this study.

Plants usually respond to environmental changes by modulating the homeostatic redox balance. This in part is usually achieved by regulating the intracellular ROS and reactive nitrogen species, especially H_2_O_2_ and NO. APX being a key enzyme in the defense system of plants, detoxifies H_2_O_2_ into water using ascorbate as an electron donor [34]. Overexpression of APX can enhance *Phytophthora nicotianae* resistance in pepper [35], blast resistance in rice [36], and *Pseudomonas syringae* pv. tabaci resistance in tobacco [37]. The APX activity in resistant grape cultivar was significantly higher than that in susceptible cultivar after inoculation with *P. viticola* [38]. Our finding is that transient expression of VvAPX1 results in an increased resistance to *P. vitocola*. Cys-32 plays a key role in APX activity [21]. The mutation of Cys-32 in VvAPX1 resulted in loss of APX enzyme activity and did not improve *N. benthamiana* resistance to *P. capsici* (Supplementary Figure S4). These results indicate that APX activity plays important role in plant immunity.

More and more leucine-rich repeat receptor-like kinases (LRR-RLKs) have been found to play important roles in development, immunity, and symbiosis [39]. Here we found that APX1 is a novelty interacting protein of BKI1 which has been reported as negative regulator of BR signaling pathway in Arabidopsis [7]. In Arabidopsis, BKI1 interacting with BRI1 and ERECTA (ER) has been reported to play an important role growth and development. MAKR2, another of membrane kinase regulator, interacting with TMK1(TRANSMEMBRANE KINASE1), functions in the root gravitropism. BRI1, ER, and TMK1 belong to LRR-RLKs. BKI1 and MAKR2 act as negative regulator of them [7,11,40]. In this study VvBKI1 and VvAPX1 transgenic Arabidopsis plants showed an enhanced resistance to oomycetes and their overexpression resulted in an increase in APX activity suggesting that these two proteins might function together to restrict pathogenesis. VvBKI1 and VvAPX1 can enhance grape resistance to *P. viticola*, *N. benthamiana* resistance to *P. capsici*, and Arabidopsis resistance to *H. arabidopsidis*. This suggests a conserved immune response that positively regulates plant resistance to oomycetes. Future studies are required to further understand the molecular mechanism that results in this resistance. Our preliminary findings of the interaction between APX and BKI1 may provide a new perspective for exploring the functions of MAKRs.

Based on our findings, we propose a hypothetical model describing the interaction between VvBKI1 and VvAPX1 in plant resistance to oomycetes (Supplementary Figure S5). Ascorbate peroxidase VvAPX1, which decomposes hydrogen peroxide, is involved in ROS metabolism in plant cells. We propose that the kinase inhibitor, VvBKI1 improves plant APX activity by interacting with VvAPX1. BKI1 which has already been reported to interact with LRR-RLK such as BRI1 and ERECTA, may also interact with an unknown RLK, that is involved in ROS perception. BKI1 may improve plant APX activity to regulate ROS signaling in cells that may further improve plant resistance against pathogens. Since genetic manipulation of grapes is not easy, model plants such as Arabidopsis and *N. bethamiana* can be used to look for the unknown LRR-RLK that interacts with BKI1 and to further understand the underlying mechanism on how this complex results in an improved resistance.

## 4. Materials and Methods

### 4.1. Plasmid Construction

*VvBKI1* and *VvAPX1* were cloned from *Vitis vinifera* ‘Thompson Seedless’, and ligated to pLB vector (Tiangen, VT206, Beijing, China) for sequencing. After sequence validation, they were amplified and inserted to pHB-GFP\PHB-Flag for transient and stable transformation. For pull down assay VvBKI1 was inserted to vector pET30a, while VvAPX1 was inserted to pCold III.

The primers used in the assay are listed in Table S2.

### 4.2. Plant Materials and Growth Conditions

*Vitis vinifera* (grape) and *Nicotiana benthamiana* (tobacco) were grown in a green house at 25 °C under 16 h light/8 h dark cycles. *Arabidopsis thaliana* (Arabidopsis) was grown in a green house at 22 °C under 16 h light/8 h dark cycles. All Arabidopsis in this study is Col-0 background. Seeds of *apx1-2* (SALK_000249C) and *apx1-6* (SALK_088596C) were requested from AraShare (a non-profit Arabidopsis share center, http://www.arashare.cn, accessed on 31 May 2021).

### 4.3. Gene Transcription Analysis by qRT-PCR

Total RNA was extracted with plant RNA kit (OMEGA Bio-Tek Inc, R6827, Norcross, GA, USA) following the instruction. cDNA was obtained from TransScript One-Step gDNA Removal and cDNA Synthesis SuperMix (TransGen Biotech, AT311-03, Beijing, China) by random primer follow the manual instruction. qPCR was performed with Hieff qPCR SYBR Green Master Mix (Yeasen, 11201ES03, Shanghai, China) in with a Bio-Rad CFX-96. Relative expression was analyzed with the BioRad (Hercules, CA, USA) CFX Manager. Primers used in the assay are listed in Table S3.

### 4.4. Transient Transformation of Grapevine Leaves

*Agrobacterium tumefacien* (GV3101) containing recombinant plasmid was cultured by liquid Luria–Bertani (LB) medium containing 50 mg/L rifampicin and 50 mg/L kanamycin. Bacterium was collected by centrifugation, resuspend by transformation buffer II (10 mM MgCl_2_, 10 mM MES, pH5.6), and adjusted OD600 = 0.4. The grape leaves were immersed in bacterial fluid and then a vacuum was pumped two or three times. Then grapes were cultured in the dark for at least 4 days. *V. vinifera* ‘Thompson Seedless’, a species susceptible to downy mildew, was used for transformation.

### 4.5. Pathogen Assay

*Plasmapora viticola* isolate ‘GX5-12’ was originally isolated as a single sporangiophore from Disu County, Guangxi province, China. It is virulent to *V. vinifera* ‘Thompson Seedless’. Separation of this isolate was performed as previously described [41]. *Plasmapora viticola* was inoculated on the leaves as described previously [4]. *P. viticola* ‘GX5-12’ was cultured for about 9 days, then suspended in ddH_2_O before use. Spore concentration of suspension was 10^5^/mL. A total of 20 μL suspension was inoculated on to the grape leaf discs 4 days after agroinfiltration. Photos were taken at 7 dpi, and the number of spores was counted as follows. The spores of 6 discs were suspended with 1 mL ddH_2_O, then placing on hemocytometer. Spores were counted under microscope (Leica DM2500, Mannheim, Germany).

*Phytophthora capsici* LT263 inoculation was performed as previously described [42]. The *P. capsici* was cultured on oat solid medium under room temperature. Agar discs (5 mm diameter) were inoculated on *N. benthamiana* leaves 24 h after infiltration. Photos were taken at 60 hpi under UV, and the lesion areas were measured with ImageJ.

Inoculation of *Hyaloperonospora arabidopsidis* Noco2 was performed as described previously [43] with minor modifications. As an obligate pathogen, *H. arabidopsidis* is cultured on Arabidopsis leaves at 16 °C for 5 days, then suspended in sterile ddH_2_O before use. Spore concentration was adjusted to about 10^5^/mL. The suspension was sprayed to 2-week-old Arabidopsis. Photos were taken and spores were counted as follows at 4 dpi. Spores, collected from infected seedlings, were suspended with H_2_O, then placed on the Hemocytometer. Spores were counted under microscope (Leica DM2500).

### 4.6. Enzyme Activity of APX

Total enzyme activity of ascorbate peroxidase in Arabidopsis was estimated with Ascorbate peroxidase (APX) test kit (Nanjing Jiancheng, A123-1-1, Nanjing, China). Ascorbate peroxidase catalyzes the reaction of ascorbic acid (ASA) with hydrogen peroxide to oxidize ascorbic acid to monodehydroascorbic acid (MDASA). As ASA is oxidized, the absorbance at 290 nm decreases in the solution. About 0.1 g rosette leaves of four-week-old Arabidopsis, were added with 900 μL R1 buffer, and homogenized in ice water bath. Centrifugation took place at 10,000 rpm for 10 min and the supernatant was then collected for analysis. A total of 100 μL extract were added with 700 μL R1 buffer, 100 μL R2 buffer, 100 μL R3 buffer. The mixture was placed into ultraviolet spectrophotometer (Thermo Fisher Scientific, GENESYS 10S, Waltham, MA, USA), the absorbance at 10 and 130 s were recorded. The protein concentration of extract was determined by Bradford reagent. For each test, leaves of same position were selected from 4-week-old Arabidopsis.

### 4.7. Protein Interaction

For immunoprecipitation–mass spectrometry (IP-MS), MYC-BKI1 was transient expressed in leaves of *N. benthamiana*. Leaves were ground to a fine powder in liquid nitrogen and solubilized with GETN buffer (10% (*v*/*v*) glycerol, 2 mM EDTA, 50 mM Tris-HCl, 150 mM NaCl; pH 7.5) containing 0.2% (*v*/*v*) Triton-X-100 and protease inhibitor cocktail (APExBIO, K1007, Houston, TX, USA). The extracts were centrifuged at 13,200 rpm for 30 min and the supernatant was incubated with anti-MYC magnetic beads (Bimake, B26301, Houston, TX, USA) for 4 h. The immunoprecipitated proteins were resuspended with 1.5× SDS loading buffer. LC-MS/MS analysis was performed using a Shimadzu LC-20AD model nanoliter liquid chromatograph coupled to an ESI tandem mass spectrometer: Triple TOF 5600 (SCIEX, Framingham, MA, USA).

For bimolecular fluorescence complementation (BiFC) assay, the CDS of VvBKI1 was cloned into pXY104, while VvAPX1 was cloned into pXY106. The plasmids were transformed into *Agrobacterium tumefacien* (GV3101). VvBKI1 and VvAPX1 were co-infiltrated into *N. benthamiana* leaves. Fluorescence signals were detected 48 h after infiltration, with a confocal microscope (Leica TCS SP5II, Mannheim, Germany).

For the Co-immunoprecipitation (CoIP) assay, *Agrobacterium tumefacien* containing pHB-VvAPX1-Flag was co-infiltrated with *Agrobacterium tumefacien* containing pHB-VvBKI1-GFP or pHB- GFP into *N. benthamiana* leaves. Total protein was extracted 2 days after infiltrating with GETN buffer (10% (*v*/*v*) glycerol, 2 mM EDTA, 50 mM Tris-HCl, 150 mM NaCl; pH 7.5) containing 0.2% (*v*/*v*) Triton-X-100, protease inhibitor cocktail (APExBIO, K1007) and phosphatase inhibitor cocktail (Yeason, 20109ES05). The same amount of total protein was incubated with anti-Flag magnetic beads (20 μL/sample; Sigma, M8823, St Louis, MO, USA) on a rotator at 4 °C for 2 h. The beads were washed 4 times with GETN buffer added 0.1%Triton-X-100 and boiled with 1.5× SDS loading buffer. The immunoprecipitation was tested by western with anti-GFP (TransGen Biotech, HT801-01) and anti-Flag (Sigma, F3165) antibody.

For pull-down assay, His-GFP, His-VvBKI1, and Glutathione S-transferase (GST)-VvAPX1 fusion protein were expressed in *E. coil* BL21 (DE3) (Shanghai Weidi biotechnology, EC1002). The GST-VvAPX1 was first incubated with glutathione sepharose 4B beads (GE Healthcare, 17-0756-01) in PBS containing 100 μM PMSF on a rotator at 4 °C for 2 h. The beads were washed three times with PBS. Equal amounts of His-GFP/His-VvBKI1 were added, and incubation on a rotator at 4 °C for 1 h. The beads were boiled in 1.5× SDS loading buffer at 100 °C for 5 min after being washed four times with PBS. A Western blot was performed using anti-His antibody (TransGen Biotech, HT501-01).

## Data Availability

All data generated by this study is available upon request.

## Supplementary Materials

The supporting information can be downloaded at: https://www.mdpi.com/article/10.3390/ijms24065106/s1.
