# Supplementary material for: Kinase Inhibitor VvBKI1 Interacts with Ascorbate Peroxidase VvAPX1 Promoting Plant Resistance to Oomycetes
Source: Int J Mol Sci. 2023 Mar 7;24(6):5106. doi: 10.3390/ijms24065106 (PMC10049515; doi:10.3390/ijms24065106)
Supplement: Supplementary file 1 [file ijms-24-05106-s001.zip › Supplementary Figure S1.pptx]

## Slide 1
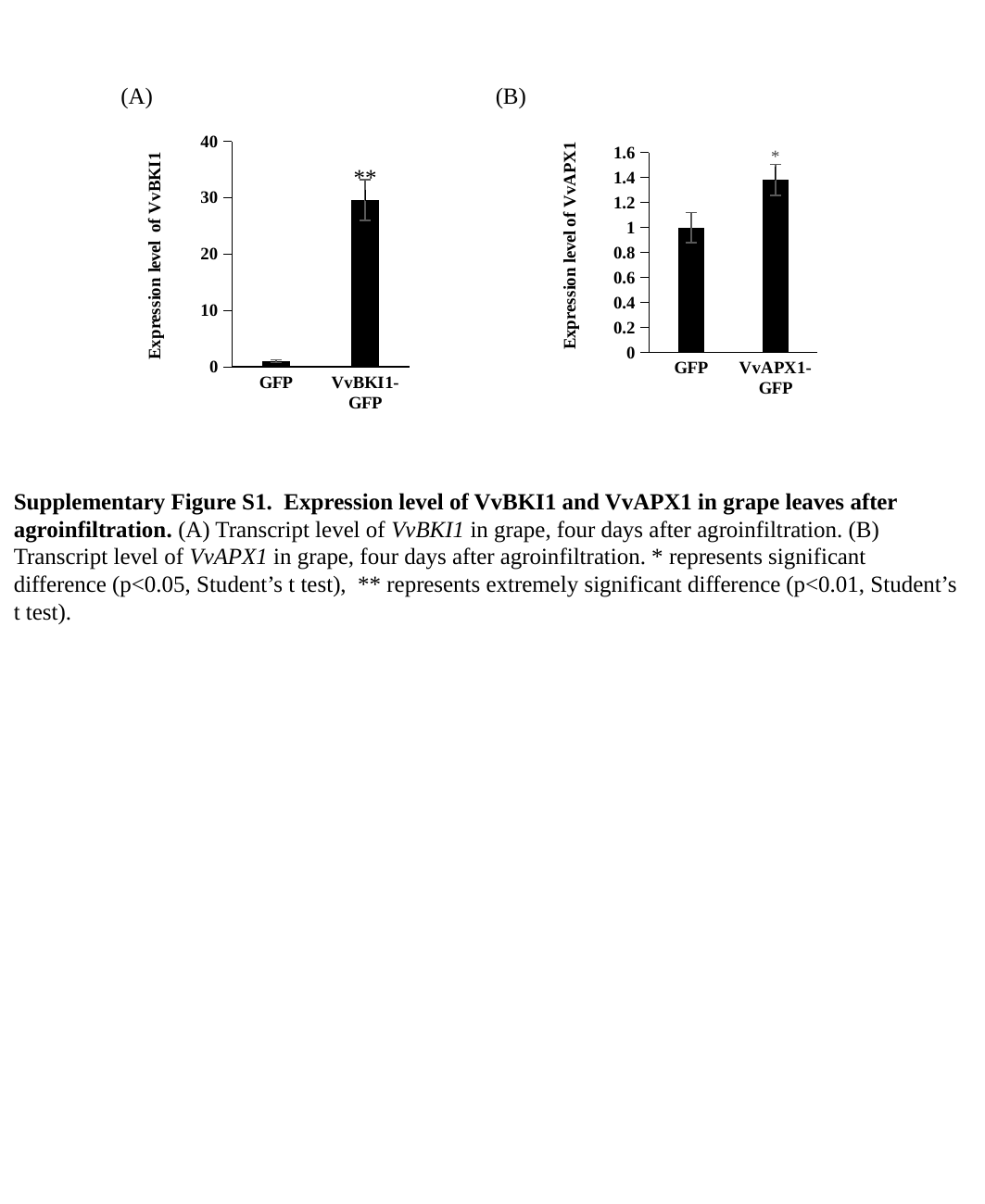

(B)
(A)
### Chart
| Category | |
|---|---|
| GFP | 1.0 |
| VvAPX1-GFP | 1.38215 |
### Chart
| Category | |
|---|---|
| GFP | 1.0 |
| VvBKI1-GFP | 29.61556 |Supplementary Figure S1. Expression level of VvBKI1 and VvAPX1 in grape leaves after agroinfiltration. (A) Transcript level of VvBKI1 in grape, four days after agroinfiltration. (B) Transcript level of VvAPX1 in grape, four days after agroinfiltration. * represents significant difference (p<0.05, Student’s t test), ** represents extremely significant difference (p<0.01, Student’s t test).
