# Supplementary material for: Kinase Inhibitor VvBKI1 Interacts with Ascorbate Peroxidase VvAPX1 Promoting Plant Resistance to Oomycetes
Source: Int J Mol Sci. 2023 Mar 7;24(6):5106. doi: 10.3390/ijms24065106 (PMC10049515; doi:10.3390/ijms24065106)
Supplement: Supplementary file 1 [file ijms-24-05106-s001.zip › Supplementary Figure S2.pptx]

## Slide 1
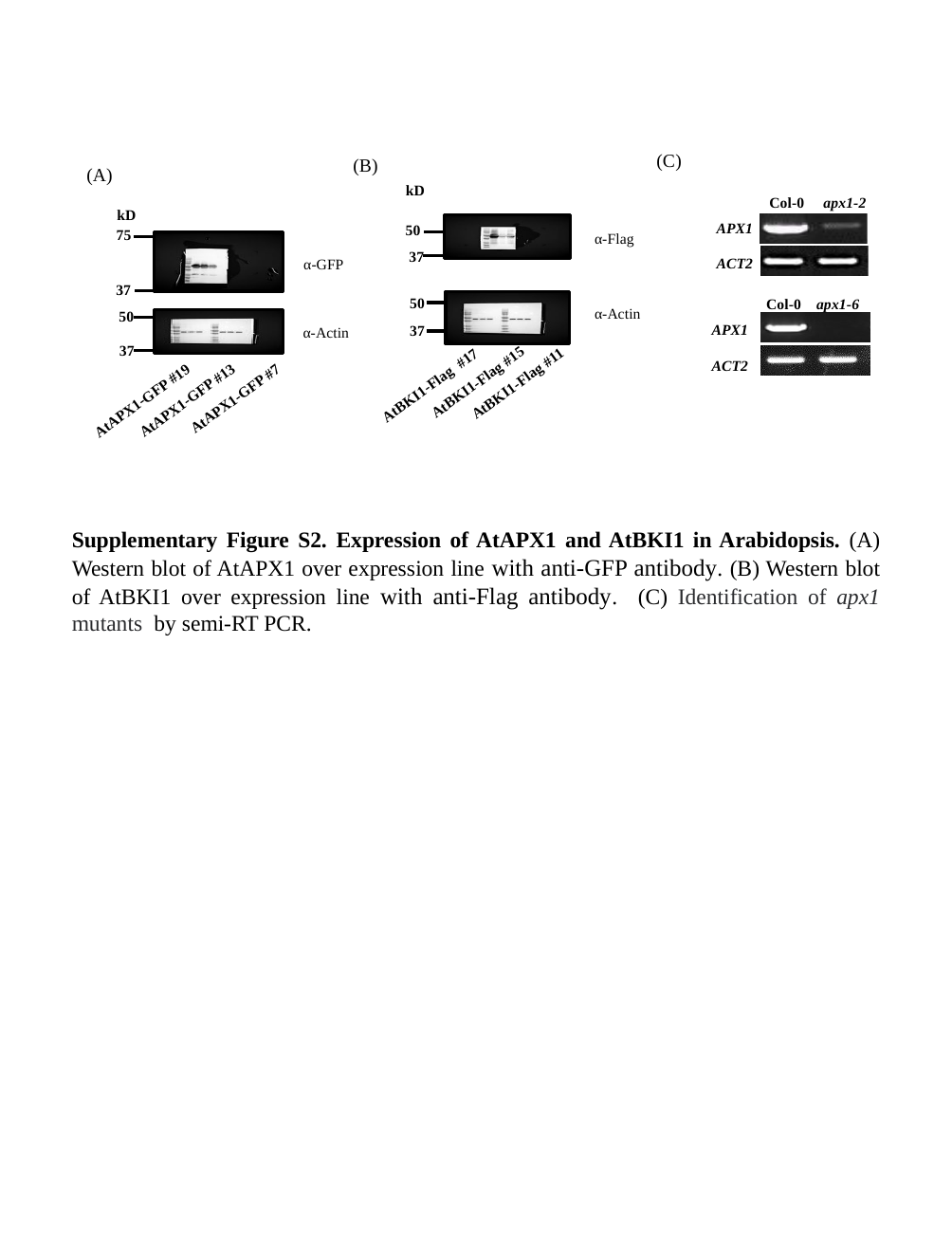

(C)
(B)
(A)
kD
50
α-Flag
37
50
α-Actin
37
AtBKI1-Flag #15
AtBKI1-Flag #11
AtBKI1-Flag #17
Col-0 apx1-2
APX1
ACT2
kD
75
α-GFP
37
50
α-Actin
37
AtAPX1-GFP #7
AtAPX1-GFP #13
AtAPX1-GFP #19
Col-0 apx1-6
APX1
ACT2
Supplementary Figure S2. Expression of AtAPX1 and AtBKI1 in Arabidopsis. (A) Western blot of AtAPX1 over expression line with anti-GFP antibody. (B) Western blot of AtBKI1 over expression line with anti-Flag antibody. (C) Identification of apx1 mutants by semi-RT PCR.
