# Supplementary material for: Kinase Inhibitor VvBKI1 Interacts with Ascorbate Peroxidase VvAPX1 Promoting Plant Resistance to Oomycetes
Source: Int J Mol Sci. 2023 Mar 7;24(6):5106. doi: 10.3390/ijms24065106 (PMC10049515; doi:10.3390/ijms24065106)
Supplement: Supplementary file 1 [file ijms-24-05106-s001.zip › Supplementary Figure S3.pptx]

## Slide 1
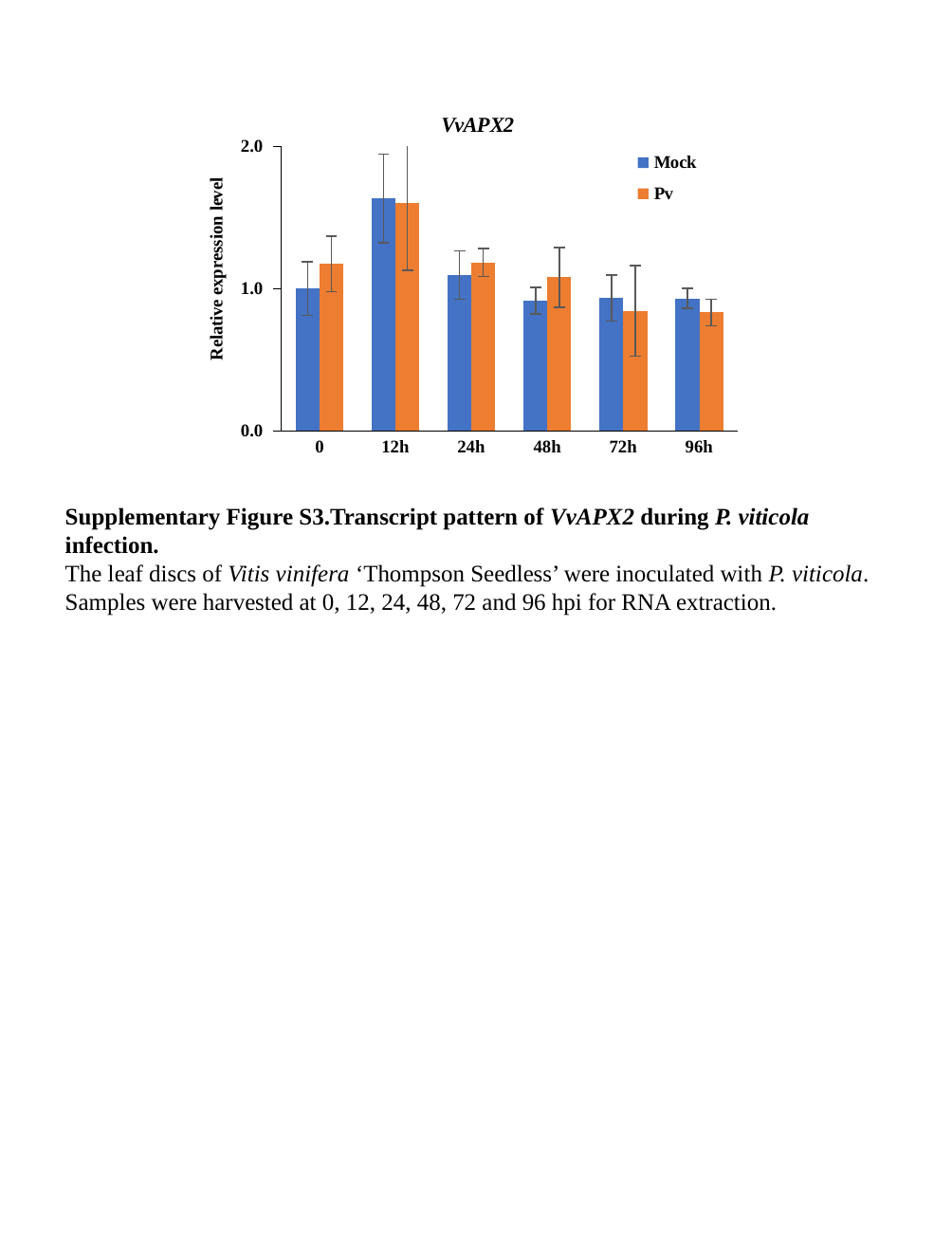

### Chart: VvAPX2
| Category | Mock | Pv |
|---|---|---|
| 0 | 1.0 | 1.17210042097789 |
| 12h | 1.63347905549574 | 1.59981621596548 |
| 24h | 1.09577272380361 | 1.18381214336566 |
| 48h | 0.914438470469457 | 1.07865130633592 |
| 72h | 0.932396133186799 | 0.842411184260683 |
| 96h | 0.929877464454793 | 0.832021353760015 |Supplementary Figure S3.Transcript pattern of VvAPX2 during P. viticola infection.
The leaf discs of Vitis vinifera ‘Thompson Seedless’ were inoculated with P. viticola.
Samples were harvested at 0, 12, 24, 48, 72 and 96 hpi for RNA extraction.
