# Supplementary material for: Kinase Inhibitor VvBKI1 Interacts with Ascorbate Peroxidase VvAPX1 Promoting Plant Resistance to Oomycetes
Source: Int J Mol Sci. 2023 Mar 7;24(6):5106. doi: 10.3390/ijms24065106 (PMC10049515; doi:10.3390/ijms24065106)
Supplement: Supplementary file 1 [file ijms-24-05106-s001.zip › Supplementary Figure S4.pptx]

## Slide 1
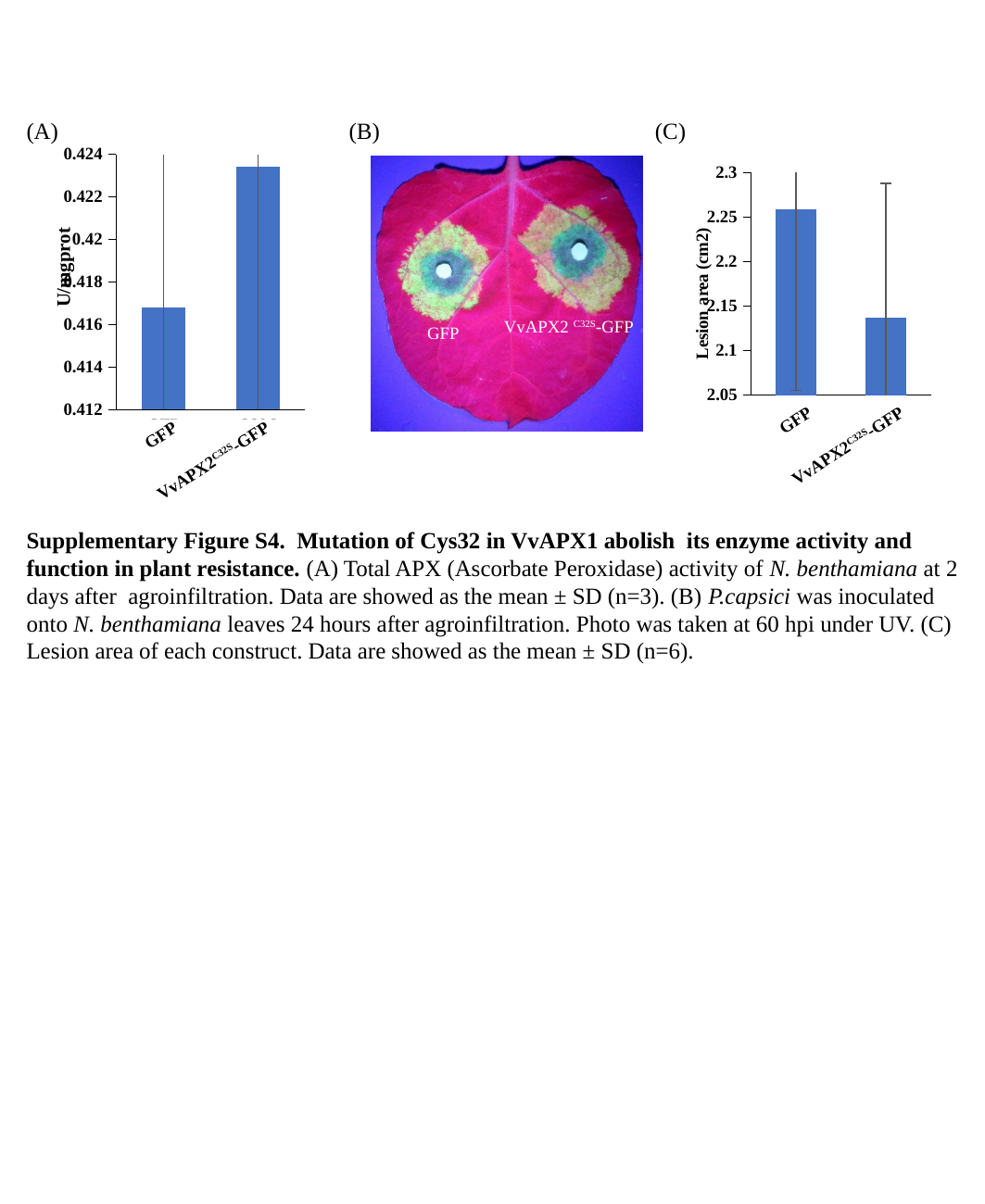

(B)
(C)
(A)
### Chart
| Category | |
|---|---|
| GFP | 0.4168185015391019 |
| C32S | 0.4234012432875305 |GFP
VvAPX2C32S-GFP
VvAPX2 C32S-GFP
GFP
### Chart
| Category | |
|---|---|
| GFP | 2.2590000000000003 |
| C32S | 2.137333333333333 |GFP
VvAPX2C32S-GFP
Supplementary Figure S4.  Mutation of Cys32 in VvAPX1 abolish its enzyme activity and function in plant resistance. (A) Total APX (Ascorbate Peroxidase) activity of N. benthamiana at 2 days after agroinfiltration. Data are showed as the mean ± SD (n=3). (B) P.capsici was inoculated onto N. benthamiana leaves 24 hours after agroinfiltration. Photo was taken at 60 hpi under UV. (C) Lesion area of each construct. Data are showed as the mean ± SD (n=6).
