# Supplementary material for: Kinase Inhibitor VvBKI1 Interacts with Ascorbate Peroxidase VvAPX1 Promoting Plant Resistance to Oomycetes
Source: Int J Mol Sci. 2023 Mar 7;24(6):5106. doi: 10.3390/ijms24065106 (PMC10049515; doi:10.3390/ijms24065106)
Supplement: Supplementary file 1 [file ijms-24-05106-s001.zip › Supplementary Figure S5.pptx]

## Slide 1
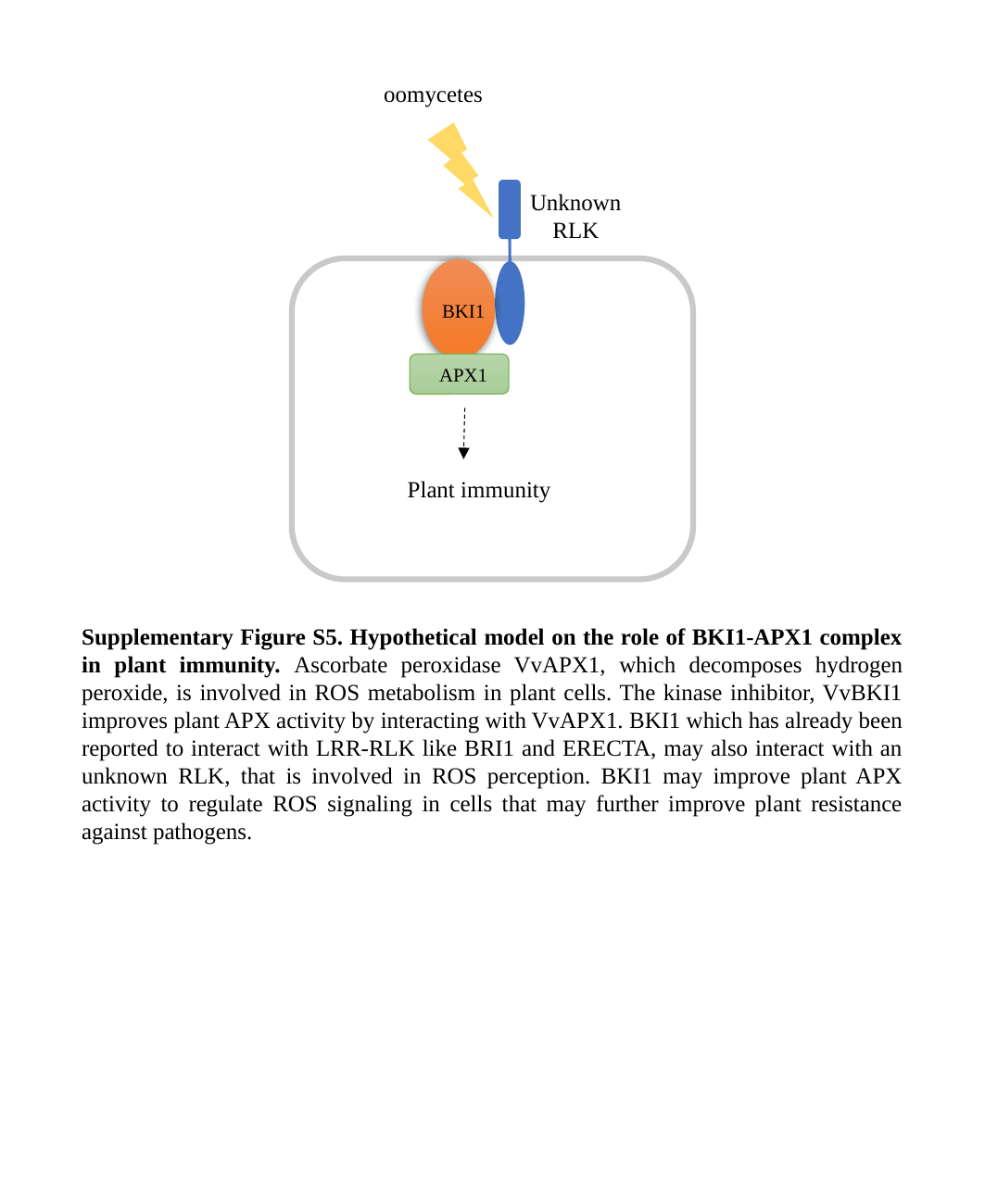

oomycetes
Unknown RLK
BKI1
APX1
Plant immunity
Supplementary Figure S5. Hypothetical model on the role of BKI1-APX1 complex in plant immunity. Ascorbate peroxidase VvAPX1, which decomposes hydrogen peroxide, is involved in ROS metabolism in plant cells. The kinase inhibitor, VvBKI1 improves plant APX activity by interacting with VvAPX1. BKI1 which has already been reported to interact with LRR-RLK like BRI1 and ERECTA, may also interact with an unknown RLK, that is involved in ROS perception. BKI1 may improve plant APX activity to regulate ROS signaling in cells that may further improve plant resistance against pathogens.
