# Supplementary material for: Kinase Inhibitor VvBKI1 Interacts with Ascorbate Peroxidase VvAPX1 Promoting Plant Resistance to Oomycetes
Source: Int J Mol Sci. 2023 Mar 7;24(6):5106. doi: 10.3390/ijms24065106 (PMC10049515; doi:10.3390/ijms24065106)
Supplement: Supplementary file 1 [file ijms-24-05106-s001.zip › Supplementary Table S1.pdf]

Supplementary Table S1 Peptide of NbAPX1 identified in LC-MS

PeptideSeqs	Protein_Qscore	iBAQ	Protein_ID
ALLSDPAFRPLVEK	2.88	133.72	tr|Q40589|Q40589_TOBAC
