# Supplementary material for: Kinase Inhibitor VvBKI1 Interacts with Ascorbate Peroxidase VvAPX1 Promoting Plant Resistance to Oomycetes
Source: Int J Mol Sci. 2023 Mar 7;24(6):5106. doi: 10.3390/ijms24065106 (PMC10049515; doi:10.3390/ijms24065106)
Supplement: Supplementary file 1 [file ijms-24-05106-s001.zip › Supplementary Table S2.pdf]

Supplementary Table S2. List of primers used in Construction

BglII-VvAPX1-F	GAAGATCTATGGGAAAGGCTTACCCTATTG
XbaI-VvAPX1-R	GCTCTAGAGCATCCAACTTCTGAGAGCTTC
EcoRI-VvAPX1-F	CGGAATTCATGGGAAAGGCTTACCCTATTG
SalI-VvAPX1-R	ACGCGTCGACTCAGCATCCA ACTTCTGAGAG
BamHI-VvBKI1-F	CGGGATCCATGGATACCGTACAGCAG
SalI-VvBKI-R	ACGCGTCGACGTCTTTGCATTTGAGTTTCTCTTC
XbaI-VvBKI-R	GCTCTAGAGTCTTTGCATTTGAGTTTCTCTTC

Red font indicates corresponding restriction enzyme recognition sites or homologous sequences of vector terminal in the primers.
