# Supplementary material for: Kinase Inhibitor VvBKI1 Interacts with Ascorbate Peroxidase VvAPX1 Promoting Plant Resistance to Oomycetes
Source: Int J Mol Sci. 2023 Mar 7;24(6):5106. doi: 10.3390/ijms24065106 (PMC10049515; doi:10.3390/ijms24065106)
Supplement: Supplementary file 1 [file ijms-24-05106-s001.zip › Supplementary Table S3.pdf]

**Supplementary Table S3.** List of primers used in qRT-PCR

| | |
|---|---|
| VvActin-F | AGACAGGATGAGCAAGGAAATC |
| VvActin-R | GCCTCCAATCCATACGCTATAC |
| VvBKI1-F | CTTCAGAGGGAGGAGGGACA |
| VvBKI1-R | GTTGCAGTTGCCACGAGAAG |
| VvAPX1-F | TGTTGTTGAAGTTAGAATCATAGCC |
| VvAPX1-R | TCGGAGCATAATCGGAGCAC |
| VvAPX2-F | GCCATCAGACAAGGCTCTCC |
| VvAPX2-R | CCTCAGCATCAGCAAATCCAAG |
| VvPR2-F | CTCCAAAGAAGCCTGGAGGG |
| VvPR2-R | CCACGAGGCTACAAACCCTT |
